# Lignin/alginate biomaterials as a promising complementary approach in ocular chlamydial infection

**DOI:** 10.3389/fcimb.2026.1843021

**Published:** 2026-06-29

**Authors:** Ana Kovacevic, Anna Pfundner, Viktoria Fischer, Ivana Lukic, Veronika Mrkus, Radmila Miljkovic, Stefan Radosavljevic, Tamara Weinmayer, Nora Geissler, Dragica Spasojevic, Miljan Baric, Ksenija Radotic, Irma Schabussova, Ursula Wiedermann, Marijana Stojanovic, Aleksandra Inic-Kanada

**Affiliations:** 1 Institute of Virology, Vaccines and Sera – Torlak, Belgrade, Serbia; 2 Institute of Specific Prophylaxis and Tropical Medicine, Center for Pathophysiology, Infectiology and Immunology, Medical University of Vienna, Vienna, Austria; 3 Institute for Multidisciplinary Research, National Institute of the Republic of Serbia, University of Belgrade, Belgrade, Serbia; 4 Institute for Biological Research “Siniša Stanković”, National Institute of the Republic of Serbia, University of Belgrade, Belgrade, Serbia

**Keywords:** *Chlamydia trachomatis*, chlamydial infection, dehydrogenation polymer lignin, guinea pig model, treatment

## Abstract

**Background:**

Ocular infection with *Chlamydia trachomatis* remains the leading infectious cause of preventable blindness worldwide. While antibiotic-based strategies have significantly reduced disease burden, sustained control in endemic regions still relies on repeated treatments, highlighting the need for complementary, locally acting approaches that reduce treatment burden and systemic exposure.

**Methods:**

We evaluated a lignin-based biomaterial formulation, dehydrogenation polymer lignin in alginate (DHP/Alg), in ocular systems *in vitro* and *in vivo*. Anti-chlamydial activity was first assessed in human ocular epithelial (IM-HConEpiC) cells infected with *C. trachomatis* serovar B. *In vivo* efficacy was then tested in a guinea pig model of ocular infection using *Chlamydia caviae*. Bacterial burden was quantified by qPCR and by enumeration of chlamydial inclusions in cell culture, while disease progression was monitored using clinical pathology scoring.

**Results:**

DHP/Alg treatment significantly reduced chlamydial inclusion numbers in ocular epithelial cells *in vitro* in a dose-dependent manner without inducing cytotoxicity. In contrast, no reduction in bacterial load was observed *in vivo* at any time point. However, a transient improvement in pathology scores was detected in animals receiving a high infectious dose, suggesting an effect on host responses rather than direct bacterial clearance. Both qPCR and culture-based measurements yielded consistent results across treatment groups.

**Conclusions:**

These findings demonstrate that, although DHP/Alg retains anti-chlamydial activity *in vitro*, these effects did not translate into a measurable reduction in bacterial burden *in vivo* under the conditions tested. Instead, the formulation appears to reduce disease severity, particularly at high pathogen loads. Together, these observations suggest that lignin-based biomaterials may exert their effects primarily at the host–pathogen interface and support further investigation of their use as locally acting adjuncts to, rather than replacements for, conventional antibiotic therapy.

## 1 Introduction

Ocular infection with *Chlamydia trachomatis* remains the leading infectious cause of preventable blindness worldwide (WHO, [[NoYear]]; Solomon et al., 2022). Repeated conjunctival infections drive chronic inflammation and progressive fibrotic scarring, ultimately impairing vision (Burton et al., 2015; Rajić et al., 2017). The WHO SAFE strategy (Surgery, Antibiotics, Facial cleanliness, and Environmental improvement) has substantially reduced global trachoma prevalence and represents a major public health success (Ageed and Khan, 2024). However, sustained control in endemic regions continues to rely heavily on repeated antibiotic administration, and reinfections remain common. While clinically stable antimicrobial resistance in *C. trachomatis* has not been demonstrated (O'Brien et al., 2019), the continued reliance on repeated mass antibiotic administration highlights the need to explore complementary, locally acting approaches that may reduce treatment burden and systemic exposure.

Biomaterial-based strategies represent a therapeutic platform that can be applied directly at the site of infection. Depending on their design, they may provide antimicrobial activity or promote tissue repair and regeneration (Ratner and Zhang, 2020). Localized intervention offers the advantage of targeting the pathological site while minimizing systemic exposure and off-target effects. The goal is not to replace antibiotics, which remain indispensable in many clinical settings, but to complement them with targeted strategies that may reduce selective pressure and unnecessary systemic use.

The effectiveness of topical therapies at the ocular surface is often limited by rapid clearance of active compounds, driven by the eye’s protective mechanisms, including epithelial barriers and the tear film (Gaudana et al., 2010). As a result, maintaining sufficient drug concentrations at the site of action remains a key challenge. To address this, a range of biomaterial-based approaches has been explored, including hydrogels, mucoadhesive polymers, and lipid- or nanoparticle-based delivery systems designed to improve stability, retention, and local bioavailability (Ludwig, 2005; Gaudana et al., 2010; Weng et al., 2017). Materials such as chitosan, hyaluronan, carrageenans, and thermosensitive polymers (e.g., poloxamers) have shown particular promise in enhancing mucoadhesion and prolonging contact with the ocular surface (Ludwig, 2005; Gratieri et al., 2010; Bucolo et al., 2012; Inic-Kanada et al., 2018). Despite these advances, the application of such biomaterial platforms to chlamydial ocular infection remains largely unexplored.

In this context, lignin-based biomaterials represent an emerging yet still underexplored approach. Lignin, a redox-active polyphenolic biopolymer capable of interacting with biological membranes and proteins (Zhao et al., 2025), can be combined with alginate, a biocompatible carrier that enables hydration and stabilization (Lee and Mooney, 2012). The formulation of dehydrogenation polymer lignin in alginate (DHP/Alg) represents a biomaterial platform with potential utility for mucosal applications (Spasojevic et al., 2016). In our previous work, DHP/Alg reduced chlamydial infectivity while preserving epithelial cell viability in a genital *in vitro* model using the A2EN epithelial cell line infected with *C. trachomatis* serovar E (CtE) (Pfundner et al., 2025).

However, genital and ocular epithelia differ substantially in barrier organization, mucin composition, surface chemistry, and innate immune regulation (Wira and Fahey, 2004; Gipson, 2007). Moreover, ocular and genital infections are caused by distinct *C. trachomatis* serovars that exhibit tissue-specific adaptations that influence epithelial interactions and the biology of infection (Miyairi et al., 2006). Consequently, findings from the genital system cannot be directly extrapolated to ocular infection.

To address this gap, we evaluated DHP/Alg in biologically relevant ocular systems, both *in vitro* and *in vivo*. We first assessed its effects in recently described, novel human ocular epithelial cells (IM-HConEpiCs) (García-Posadas et al., 2022) infected with the clinically relevant ocular *C. trachomatis* serovar B (CtB) (Ghasemian and Holland, 2024). We then extended these analyses to an established *in vivo* guinea pig model of ocular infection using *Chlamydia caviae* (Rank and Whittum-Hudson, 1994; Belij-Rammerstorfer et al., 2015; Filipovic et al., 2017), enabling parallel assessment of bacterial burden and disease pathology.

Two principal animal models for chlamydial ocular disease have been described. Non-human primates have been used in conjunction with several ocular and non-ocular *C. trachomatis* serovars (Rank and Whittum-Hudson, 1994), whereas the guinea pig model involves ocular infection with *C. caviae*, a host-restricted natural pathogen first described by Murray (1964). Importantly, this model induces an acute conjunctivitis resembling human inclusion conjunctivitis and, upon repeated infections, can lead to a trachoma-like disease (Monnickendam et al., 1980). The guinea pig remains the only small-animal model that naturally recapitulates key features of human ocular chlamydial infection (Stein et al., 2013; Belij-Rammerstorfer et al., 2015; Inic-Kanada et al., 2015; Filipovic et al., 2017; Inic-Kanada et al., 2018; Inic-Kanada et al., 2020).

By integrating ocular *in vitro* and *in vivo* models, this study aimed to determine whether lignin-based biomaterials can influence infection outcomes under physiologically relevant conditions.

## 2 Material and methods

### 2.1 Chlamydial strains

CtB and *C. caviae* were propagated in McCoy cells under standard conditions (Caldwell et al., 1981). Infected cell monolayers were harvested at late stages of infection and mechanically disrupted with glass beads to release elementary bodies (EBs). The resulting suspension was clarified and purified by ultracentrifugation at 160,000 × g through a discontinuous Gastrografin gradient (40/44/54%). Purified EBs were recovered from the 40/44% interface, washed in phosphate-buffered saline, and resuspended in Sucrose-Phosphate-Glutamic acid (SPG) buffer. Aliquots were stored at -80 °C until use. The IFUs/mL were determined by titration in McCoy cells and were 1 × 10^7^ IFU/mL for CtB and 5 × 10^8^ IFU/mL for *C. caviae*.

### 2.2 Cell line and culture conditions

To perform the *in vitro* experiments, immortalized human conjunctival epithelial (IM-HConEpiC) cells (García-Posadas et al., 2022) were used. Cells were cultured in DMEM/Ham’s F-12 medium (SH30023.01, Cytiva, US) supplemented with 10% fetal calf serum (FCS) (9665, Sigma, US), 25 µg/mL vancomycin (0242.3, Roth, Germany), and 10 µg/mL gentamicin (G3632-25G, Sigma, US). Cells were passaged at 70–80% confluency using 0.05% trypsin/0.02% EDTA (P10-023100, Pan Biotech, Germany) and neutralized with the culture medium. Following detachment, cells were centrifuged at 400 × g for 5 min, resuspended in the same medium, and transferred to new culture flasks. For titration of ocular swab samples, McCoy cells (CRL-1696, McCoy Fibroblast Mouse, 70055443, ATCC) were used. Cells were maintained in high-glucose DMEM (D5796, Sigma-Aldrich, US) supplemented with 10% fetal calf serum (FCS) (9665, Sigma, US) and passaged as described for IM-HConEpiC cells. Following inoculation with swab suspensions, cells were cultured in DMEM/Ham’s F-12 medium (SH30023.01, Cytiva, US) supplemented with 10% FCS (9665, Sigma, US), 25 µg/mL vancomycin (0242.3, Roth, Germany), 10 µg/mL gentamicin (G3632-25G, Sigma, US), and 1 µg/mL cycloheximide (C4859, Sigma-Aldrich, US).

### 2.3 DHP/Alg preparation

DHP lignin was synthesized as previously described (Spasojevic et al., 2016) and formulated with alginate (A0682, Sigma-Aldrich, US) at a 1:2 ratio (DHP: Alg), as detailed in Pfundner et al. (2025). For stock preparation, DHP and alginate were dissolved in 100% DMSO and diluted in sterile distilled water to generate a 10 mg/mL DHP/Alg stock solution containing 5% DMSO. The stock solution was subsequently diluted in either IM-HConEpiC culture medium or sterile PBS, sterile-filtered, and adjusted to the indicated working concentrations. Final DMSO concentrations were 0.25% and 0.0375% for the 500 µg/mL *in vitro* and 75 µg/mL *in vivo* formulations, respectively. The lower concentration used for *in vivo* application was selected, in part, to minimize DMSO exposure during repeated topical ocular administration over 14 consecutive days. Based on the very low final DMSO concentrations used *in vivo* and previous reports indicating limited cytotoxic effects at concentrations ≤0.1% DMSO in many cell culture systems, a separate DMSO control was not included (Moskot et al., 2019). All concentrations stated in the manuscript refer to DHP, while the DHP: Alg ratio was consistently maintained at 1:2.

### 2.4 Cytotoxicity assay

IM-HConEpiC cells were seeded at a density of 5,000 cells/well in a 96-well plate. The next day, the medium was removed, and cells were treated with DHP/Alg (1–500 μg/mL for DHP in medium). Controls included cells without treatment, set to 100% viability, and the medium-only (no cells) control, which was subtracted from all samples (background correction). After 48 h, 10 µL of Cell Counting Kit-8 (CCK-8, 96992, Sigma-Aldrich, US) reagent was added, and the plate was incubated for 4 h at 37 °C, 5% CO2, and 95% humidity. Cell viability was measured at 450 nm using a microplate reader (Varioskan Flash, Thermo Scientific).

### 2.5 *In vitro* infection assay

IM-HConEpiC cells were seeded at 60,000 cells per well on coverslips (diameter of 12 mm) in a 24-well plate and were infected the next day with CtB (MOI of 0.5). The plate was centrifuged for 1 h to facilitate infection, then incubated for 1 h at 37 °C/5% CO_2_, and 95% humidity. The medium was removed and replaced with DHP/Alg, DHP alone, or alginate alone. Azithromycin (1 μg/mL) served as a positive control, while medium alone served as a negative control. Non-infected cells were an additional control. After 48 h, the medium was removed, and the cells were briefly washed with DPBS and fixed with ice-cold methanol at −20 °C for 10 min. Following fixation, the cells were washed again with DPBS. Coverslips were stained with 200 µL FITC-labeled anti-Chlamydia-LPS antibody (MA1 7339, Invitrogen, US), diluted 1:50 in 5% BSA in DPBS for 30 min at RT in the dark. The solution was removed, and the cells were washed 3 × 5 min with DPBS and counterstained with 1 μg/mL 4′, 6-diamidino-2-phenylindole (DAPI, D9542, Invitrogen, US). Coverslips were mounted on microscopic slides using DAKO fluorescent mounting medium (Agilent Technologies, US) and stored overnight at 4 °C. They were analyzed using the TissueFAXS imaging system (TissueGnostics, Austria), and inclusion bodies were counted in 20 systematically selected microscopic fields (40×) for semiquantitative measurement (Nagarajan et al., 2011).

### 2.6 Experimental animals

Female Hartley guinea pigs (6 weeks of age, 300–350 g) were used in this study. Each experimental group consisted of four animals per infectious dose and time point, for a total of 64 animals. Animals were housed in filter-top cages under a 12 h light/dark cycle with food and water provided *ad libitum*. Prior to inclusion, guinea pigs were pre-screened using an in-house-optimized ELISA as previously described (Belij-Rammerstorfer et al., 2015). All procedures were conducted in accordance with national regulations (Sluzbeni Glasnik No. 41/09), the Guide for the Care and Use of Laboratory Animals of the Torlak Institute, and the principles of the Basel Declaration, in adherence to the 3Rs framework (replacement, reduction, refinement). Animals were monitored daily by trained personnel, and veterinary care was provided when required. All efforts were made to minimize discomfort. Terminal euthanasia was performed by intraperitoneal injection of pentobarbital (150–200 mg/kg body weight) under deep anesthesia. No unexpected deaths or severe adverse events occurred during the study.

### 2.7 *In vivo* infection and treatment protocol

Guinea pigs were infected ocularly with 25 µL/eye of *C. caviae* suspended in SPG (day 0). The inoculum contained either a low dose of 10² IFU (Groups 1–4), 10^4^ IFU (Groups 5–8), or a high dose of 10^6^ IFU (Groups 9–12). Groups 13–16 received only 25 µL of SPG.

2 h post infection (day 0) and continuing through day 14, animals were treated once daily with 25 µL/eye of one of the following formulations: 150 µg/mL alginate in PBS (Groups 1, 5, 9, 13), 75 µg/mL DHP in PBS (Groups 2, 6, 10, 14), 75 µg/mL DHP + 150 µg/mL alginate in PBS (Groups 3, 7, 11, 15), or PBS alone (Groups 4, 8, 12, 16). The concentration of DHP/Alg used for *in vivo* treatment (75 µg/mL DHP + 150 µg/mL alginate) was selected based on tolerability considerations and previous *in vitro* studies (Pfundner et al., 2025). Animals were weighed daily and evaluated for ocular pathology using a standardized clinical scoring system (described in 2.8). Ocular swabs were collected on days 0, 4, 7, and 14, post-infection, after pathology/clinical scoring and before daily treatment.

### 2.8 Pathology scoring

In brief, ocular pathology was evaluated daily by a trained ophthalmologist who was blind to the experimental groups. The palpebral and bulbar conjunctivae were examined for signs of erythema, edema, and exudate. Clinical severity was graded on a standardized 0.5–4 scale, where 0.5 indicated a trace inflammatory response; 1, mild erythema or edema affecting either conjunctival surface; 2, clear erythema or edema of either the palpebral or bulbar conjunctiva; 3, evident involvement of both conjunctival surfaces; and 4, pronounced bilateral erythema or edema with exudate formation (Filipovic et al., 2017).

### 2.9 Histopathological analysis of conjunctival tissue

Conjunctival tissue was collected at days 7 and 14 post-infection and fixed in 1% formaldehyde in PHEM buffer (60 mM PIPES, 25 mM HEPES, 10 mM EGTA, and 2 mM MgCl_2_). Samples were paraffin-embedded, and 4 µm sections were prepared for histological analysis and routinely stained with hematoxylin and eosin (H&E). Evaluation of inflammatory and cellular infiltration was performed as previously described (Inic-Kanada et al., 2015), in a blinded manner, and five conjunctival sections per animal were analyzed. Representative images shown in the manuscript were selected from the evaluated conjunctival sections.

### 2.10 Determination of infectious burden from conjunctival swabs

Conjunctival swabs used for quantification of *C. caviae* load were obtained using the Copan Universal Transport Medium (UTM-RT) system and stored at −80 °C until processing.

#### 2.10.1 Cell culture

Infectious units (IFU) were determined by culture on McCoy cell monolayers as previously described (Filipovic et al., 2017). Briefly, swab suspensions were inoculated onto cells by centrifugation-assisted infection and incubated for 24 h before fixation with methanol. Chlamydial inclusions were detected using a FITC-conjugated monoclonal antibody (1:50) against chlamydial LPS (MA1-7339, Invitrogen, US), counterstained with 1 μg/mL 4′,6-diamidino-2-phenylindole (DAPI, D9542, Invitrogen, US), and quantified by fluorescence microscopy. Images were analyzed using the TissueFaxs microscope (TissueGnostics, Austria) with a 20x objective, and inclusion bodies were counted in 20 systematically selected microscopic fields (40×) for semiquantitative measurement to obtain IFUs/mL.

#### 2.10.2 qPCR

Additionally, DNA was extracted from swab suspensions using the QIAamp DNA Mini Kit (QIAGEN, Germany) according to the manufacturer’s guidelines, with minor modifications to the protocol. Ocular swabs were vortexed for approximately 60 s, and 500 µL of the swab transport medium was collected for processing. Samples were centrifuged at 17,000 × g for 10 min to pellet the material, and the supernatant was carefully removed. The pellet was resuspended in 180 µL Buffer ATL, and 20 µL Proteinase K was added.

Samples were incubated at 56 °C for 1 h with shaking at 350 rpm, with brief vortexing every 15 min. After incubation, samples were centrifuged, and 4 µL RNase (100 mg/mL) was added. Following pulse vortexing and a 2 min incubation at room temperature, the samples were centrifuged again. Subsequently, 400 µL Buffer AL was added, mixed by vortexing, and incubated at 70 °C for 10 min (350 rpm). After a spin-down, 200 µL of 96% ethanol was added, and the mixture was mixed thoroughly. The lysate was applied to a QIAamp Mini spin column and centrifuged at 6,000 × g for 1 min. The column was transferred to a new collection tube and washed twice with 500 µL of Buffer AW1 (centrifugation at 6,000 × g for 1 min each), followed by a wash with 500 µL of Buffer AW2 (centrifugation at 17,000 × g at 4 °C for 10 min). An additional dry spin was performed at 17,000 × g for 1 min.

DNA was eluted in 40 µL Buffer AE after incubation for 5 min at room temperature, followed by centrifugation at 6,000 × g for 1 min. This elution step was repeated once, resulting in a total elution volume of 80 µL. DNA concentration was measured using a NanoDrop spectrophotometer (Thermo Fisher Scientific, US), and samples were stored at −20 °C until further analysis.

Chlamydial load was quantified by qPCR targeting the *Chlamydiaceae*-specific 23S rRNA gene. To measure absolute pathogen burden per ocular swab, 1 µL of extracted DNA was used, ensuring the readout reflected total recovered chlamydial DNA rather than a normalized input. The following primers were used: Ch23S-F: CTG AAA CCA GTA GCT TAT AAG CGG T, Ch23S-R: ACC TCG CCG TTT AAC TTA ACT CC. Absolute quantification was achieved using a plasmid standard containing the 23S sequence, kindly provided by Dr. Hanna Marti, run as a 10-fold dilution series (10^8^–10 copies, in duplicate). Sample concentrations were calculated from the resulting standard curve and expressed as chlamydial genomes per mL of swab material, enabling comparison with fluorescence microscopy results (IFU/mL). The qPCR Mastermix was prepared using SYBR Green Supermix kit (1725271, Bio-Rad, US) and run on a CFX Duet Real-Time PCR System under the following conditions: 37 °C for 2 min, 95 °C for 10 min, followed by 45 cycles of 95 °C for 10 s and 60 °C for 30 s. Results reflect chlamydial genomes per µL of extracted DNA, which can then be extrapolated to chlamydial genomes per mL of swab suspension (×80 for elution volume; ×2 to convert from 0.5 mL to per mL).

### 2.11 Statistics

Statistical analyses were performed using GraphPad Prism v.10. For comparisons involving multiple groups, one-way or two-way ANOVA was applied as appropriate, followed by Dunnett’s or Sidak’s multiple-comparisons test. For IFU and qPCR data, values were log-transformed prior to analysis. A p-value < 0.05 was considered statistically significant. Effect sizes for selected comparisons were additionally calculated and are reported as eta squared (η²). The study was designed as an exploratory proof-of-concept study. Sample sizes were predefined based on previous experience with the guinea pig ocular infection model, expected biological variability, ethical considerations, and the aim to minimize animal use in accordance with the 3Rs principle. The relatively limited group sizes may reduce statistical power to detect moderate effect sizes and increase the risk of Type II errors, which was taken into account when interpreting the findings. Importantly, despite the exploratory design and limited group sizes, statistically significant differences were still detected in selected readouts, supporting the biological relevance of the observed effects.

## 3 Results

### 3.1 DHP/Alg does not exhibit cytotoxic effects on ocular IM-HConEpiC cells

**Figure 1:**
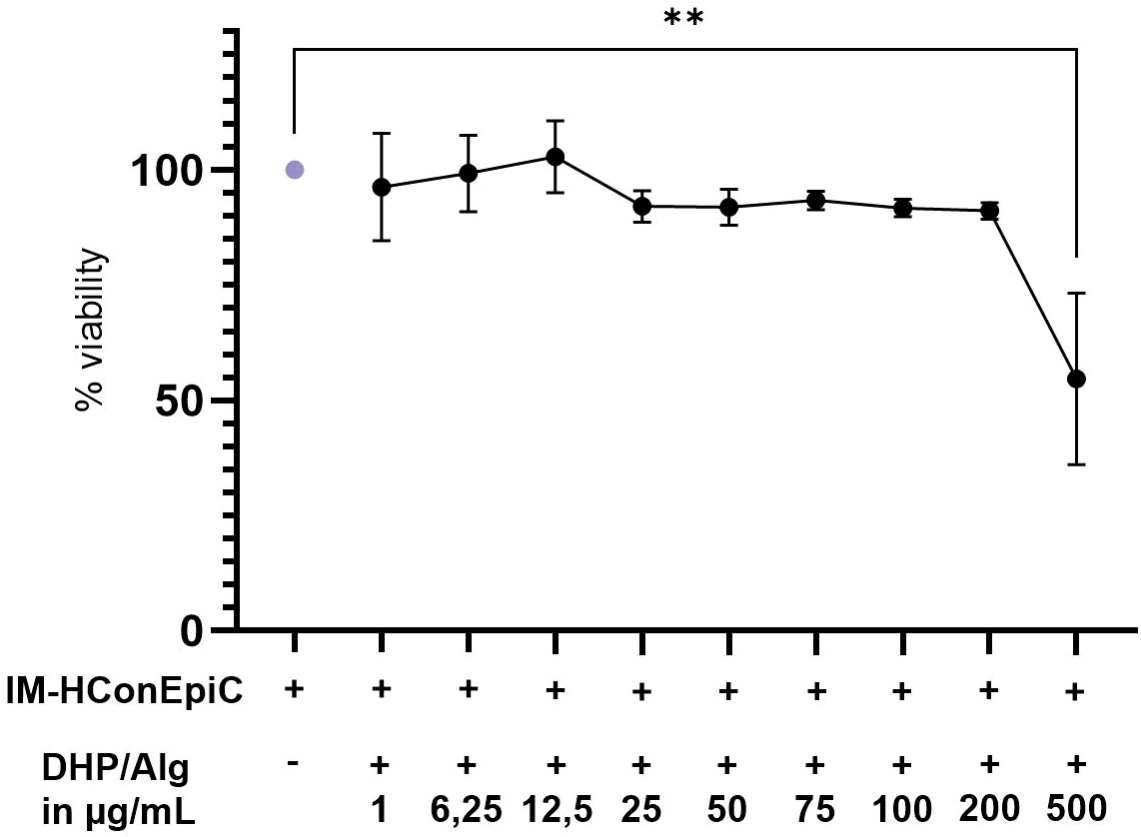
*In vitro* cytotoxicity of DHP/Alg in IM-HConEpiC cells. Cell viability was assessed following treatment with DHP/Alg (1:2 ratio) across DHP concentrations ranging from 1–500 µg/mL. Untreated cells were used as controls and set to 100% viability, with all values expressed relative to this control. The x-axis represents DHP concentration, while the y-axis indicates cell viability (%). Error bars represent the mean ± SD of three biological replicates, which were performed in technical triplicate. Statistical significance was determined using one-way ANOVA followed by Dunnett’s multiple comparisons test, comparing each condition to the untreated control. Significance levels are indicated as *p ≤ 0.05, **p ≤ 0.01, ***p ≤ 0.001, ****p ≤ 0.0001.

A viability assay was performed to assess the cytotoxic effects of DHP/Alg treatment on IM-HConEpiC cells across a range of concentrations (**Figure 1**). Cell viability was normalized to untreated controls, which were set to 100%. Across concentrations ranging from 1 to 200 µg/mL, no significant reduction in viability was observed, with all values consistently remaining above 90%, indicating good tolerability of the formulation. A significant decrease in viability was detected only at the highest concentration tested (500 µg/mL) (**p ≤ 0.01), suggesting dose-dependent cytotoxicity at high levels. Based on these results, concentrations up to 200 µg/mL were considered non-cytotoxic to ocular epithelial cells and were selected for subsequent *in vitro* experiments.

### 3.2 Treatment with DHP/Alg reduces chlamydial infection in ocular IM-HConEpiC cells

**Figure 2:**
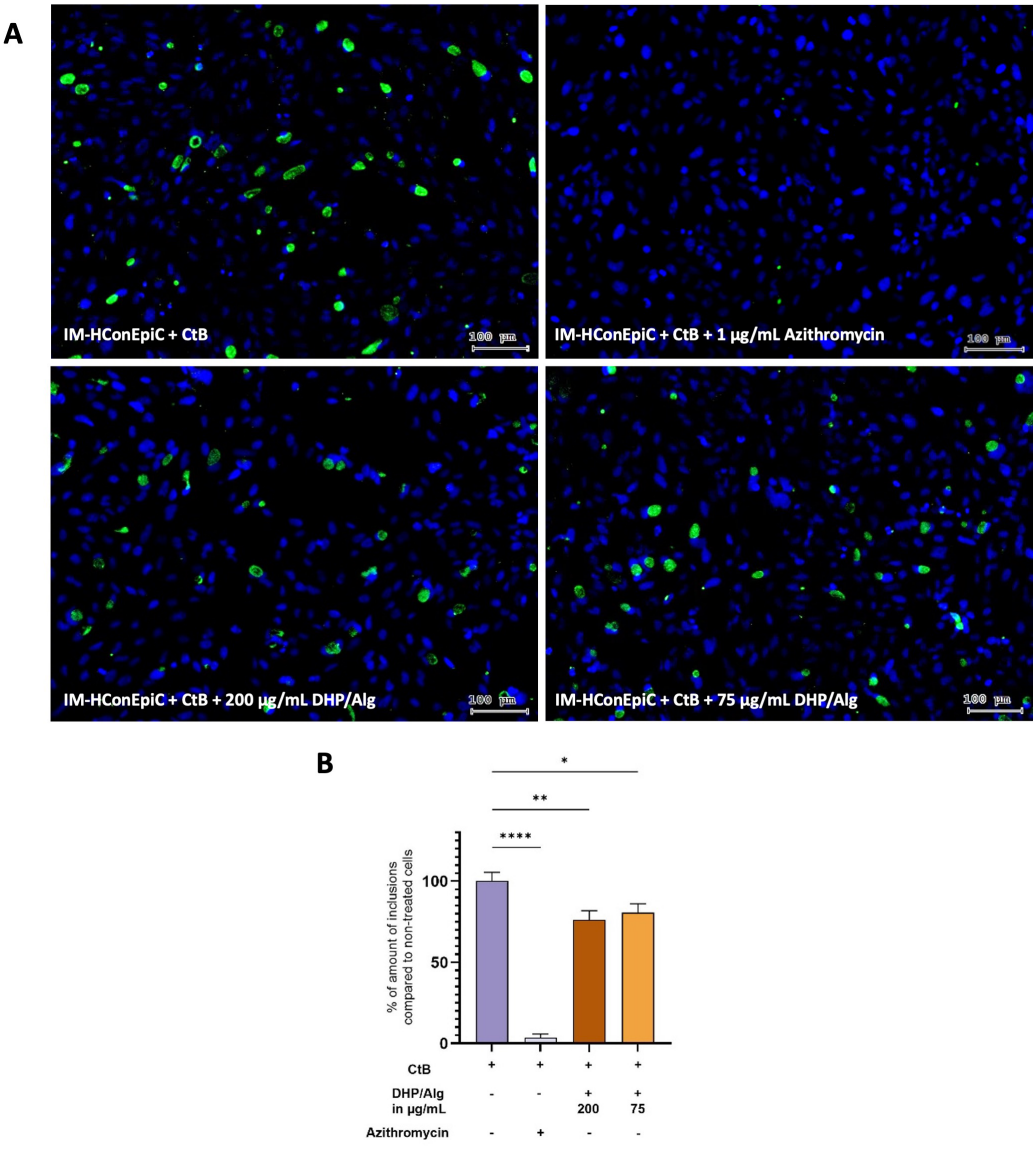
*In vitro* infection of IM-HConEpiC with CtB, MOI of 0.5, assessed by fluorescence microscopy. **(A)** Representative fluorescent images of IM-HConEpiC cells under the following conditions: non-treated CtB-infected cells; cells + CtB + azithromycin (1 µg/mL); cells + CtB + DHP/Alg (200 µg/mL); and cells + CtB + DHP/Alg (75 µg/mL). **(B)** Semiquantitative analysis of infection levels. The x-axis represents experimental conditions, while the y-axis indicates the percentage of inclusions compared to non-treated infected cells. Statistical significance was determined using one-way ANOVA followed by Dunnett’s multiple comparisons test. Significance levels are indicated as *p ≤ 0.05, **p ≤ 0.01, ***p ≤ 0.001, ****p ≤ 0.0001.

The effect of DHP/Alg on chlamydial infection was assessed using an *in vitro* infection model in IM-HConEpiC cells (**Figure 2**). Treatment with DHP/Alg (1:2 ratio) resulted in a significant reduction in the number of inclusions at 75 µg/mL DHP (p ≤ 0.05) and a more pronounced reduction at 200 µg/mL DHP (p ≤ 0.01), indicating a dose-dependent inhibitory effect. Control conditions included uninfected IM-HConEpiC cells and infected cells treated with 1 µg/mL azithromycin, which served as a positive treatment control, resulting in near-complete clearance of infection. As expected, the anti-chlamydial effect of DHP/Alg was less pronounced than that of azithromycin; however, a clear and reproducible decrease in inclusion numbers was observed. Importantly, treatment with DHP alone (75 and 200 µg/mL) or alginate alone (150 µg/mL) did not reduce infection, indicating that the anti-chlamydial effect depends on the combined DHP/Alg formulation.

### 3.3 Limited *in vivo* efficacy of DHP/Alg against ocular *C. caviae* infection

**Figure 3:**
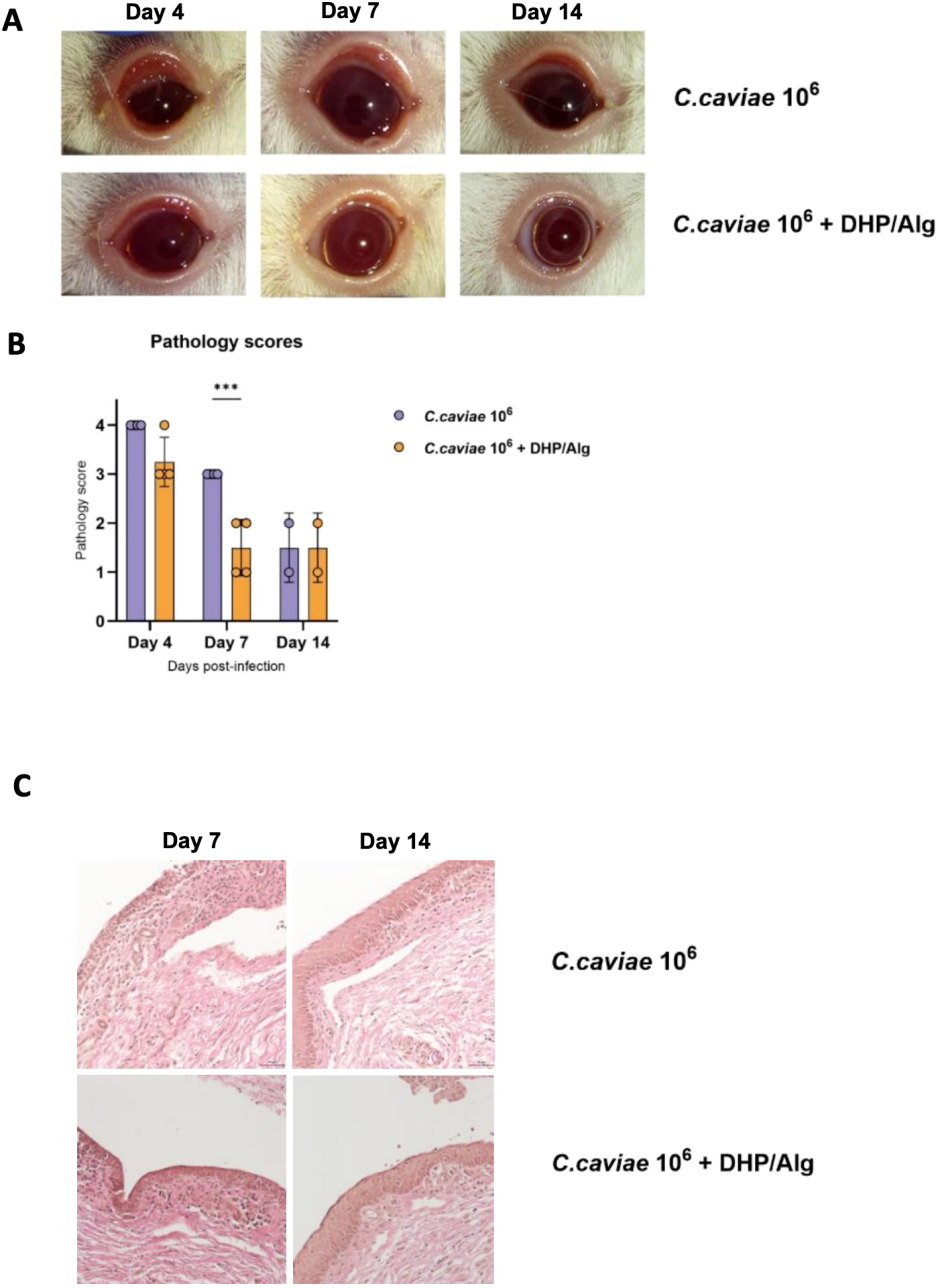
Ocular pathology scores in guinea pigs following infection with 10^6^ IFU *C. caviae*/eye, with or without DHP/Alg (75 µg/mL) treatments during the post-infection period. Each experimental group consisted of four animals per time point. **(A)** Representative photographs of guinea pigs taken on post-infection day 4, day 7, and day 14. **(B)** Ocular pathology scores recorded at post-infection day 4, day 7, and day 14 (indicated on x-axis). The y-axis shows the pathology score on a 0.5–4 scale (0.5 = a trace inflammatory response; 1 = mild erythema or edema affecting either conjunctival surface; 2 = clear erythema or edema of either the palpebral or bulbar conjunctiva; 3 = evident involvement of both conjunctival surfaces; and 4 = pronounced bilateral erythema or edema with exudate formation (Filipovic et al., 2017)). Statistical analysis was performed using two-way ANOVA followed by Sidak’s multiple comparisons test. Only statistically significant differences are shown. Significance levels are indicated as *p ≤ 0.05, **p ≤ 0.01, ***p ≤ 0.001. Effect size analysis showed that the treatment factor accounted for 11.23% of the total variation (η² = 0.1123), while the interaction effect accounted for 6.98% (η² = 0.0698). **(C)** Representative H&E-stained conjunctival sections from guinea pigs collected at 7 and 14 days post-infection. Scale bar: 50 µm.

To evaluate the *in vivo* efficacy of DHP/Alg, guinea pigs were ocularly infected with escalating doses of *C. caviae*, and disease progression was monitored daily using clinical pathology scoring. No significant differences in pathology scores were observed between treated and untreated animals in the low (10² IFU) and medium (10^4^ IFU) infection dose groups throughout the observation period. In contrast, in the high-dose group (10^6^ IFU), animals treated with DHP/Alg showed a transient reduction in pathology scores at day 7 post-infection compared to PBS-treated controls (**Figures 3A, B**). This effect was not sustained, as pathology scores converged between groups at later time points. Overall, the kinetics of disease progression were comparable across groups, with peak inflammation occurring during the early phase of infection, followed by gradual resolution. Histological examination of conjunctival tissue collected at days 7 and 14 post-infection revealed varying degrees of inflammation and epithelial remodeling (**Figure 3C**). At day 7 post-infection, DHP/Alg-treated animals generally exhibited reduced inflammatory infiltration and better preservation of conjunctival architecture compared to untreated controls. By day 14, inflammatory changes were reduced in both groups, consistent with the overall resolution phase of infection.

**Figure 4:**
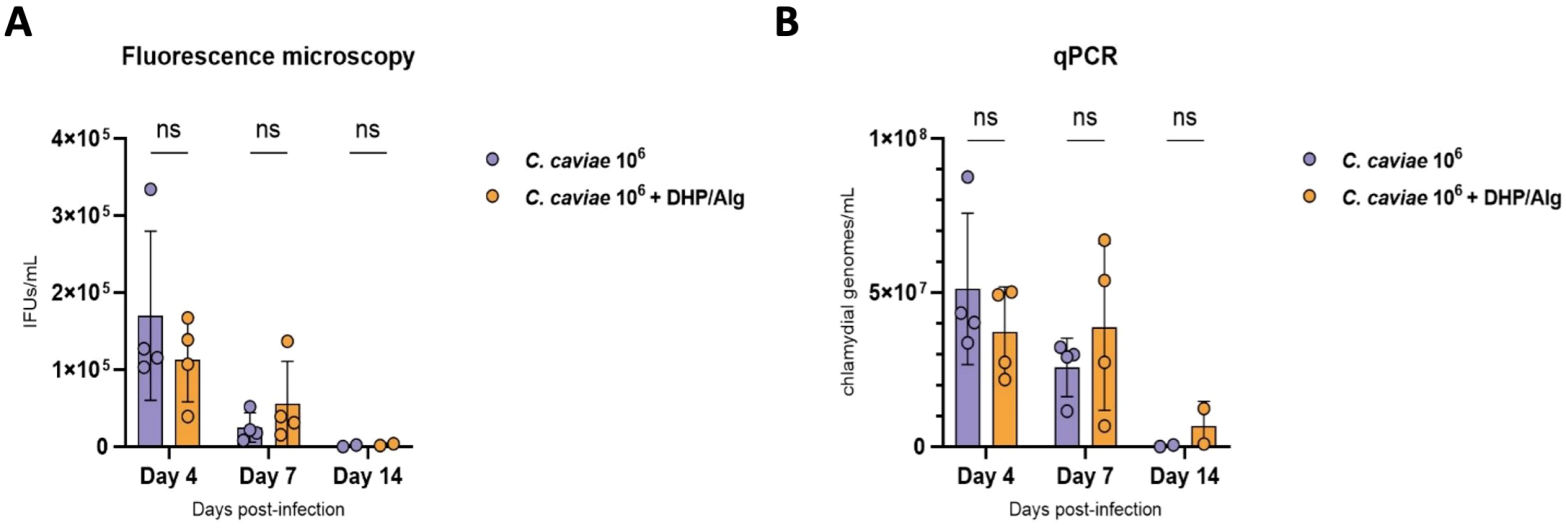
Analysis of ocular swab suspensions collected from guinea pigs infected with 10^6^ IFUs of *C. caviae*/eye and either left untreated or treated with DHP/Alg (75 µg/mL) during the post-infection period. Each data point represents an individual animal (n = 4 per group per time point). **(A)** Quantification of infectious units in ocular swab suspensions collected at post-infection day 4, day 7, and day 14, as determined by fluorescence microscopy. The y-axis indicates IFUs/mL of swab suspension. IFUs/mL were calculated by counting inclusions in 20 systematically selected microscopic fields, extrapolating to the total coverslip area, and correcting for the dilution factor. **(B)** Quantification of chlamydial load in corresponding swab samples by qPCR. Samples were collected at the same time points, and experimental groups were identical to those in **(A)**. The y-axis represents chlamydial genome copies/mL. Genome copy numbers were determined using a standard curve and subsequently converted from copies per reaction well to copies per mL of swab suspension. Statistical significance was assessed using two-way ANOVA followed by Sidak’s multiple comparisons test. Significance levels are indicated as *p ≤ 0.05, **p ≤ 0.01, ***p ≤ 0.001.

Ocular swab analysis focused primarily on animals infected with the highest dose of *C. caviae* (10^6^ IFU), as only this group showed transient differences in clinical pathology. Quantification of chlamydial burden by both fluorescence microscopy (live inclusion counts) and qPCR (**Figure 4**) revealed no significant differences between DHP/Alg-treated and untreated animals at days 4, 7, and 14 post-infection. Both methods consistently demonstrated comparable bacterial burdens across groups, indicating that DHP/Alg treatment did not significantly reduce chlamydial load *in vivo* under the conditions tested. Absolute values are shown to enable direct comparison between the two quantification methods.

Animals infected with medium and low doses (10^4^ and 10² IFU) were also analyzed, and these data are presented in **Supplementary Figure 1**. In these groups, infectious burden is expressed relative to day 4 (untreated controls), as the primary objective was to compare treatment-associated trends rather than absolute bacterial burden. No treatment-associated differences were observed.

## 4 Discussion

Our previous work demonstrated that DHP/Alg interferes with chlamydial infection dynamics in genital epithelial cells *in vitro*, primarily by reducing bacterial adhesion and intracellular load, with the strongest effect observed when applied simultaneously with elementary bodies, suggesting interference with early infection events (Pfundner et al., 2025). Although chlamydial infection shares conserved features across mucosal tissues, the genital and ocular environments represent distinct biological systems with differences in epithelial structure, immune regulation, and microbiome composition, which can critically influence infection dynamics and treatment responses (Dong et al., 2011; Aiyar et al., 2014; Ziklo et al., 2016). The present study extends these observations to the ocular setting by employing a physiologically relevant and recently characterized immortalized human conjunctival epithelial cell line (IM-HConEpiC), which retains key features of conjunctival epithelium, including mucin production, epithelial junctional integrity, and responsiveness to oxidative and inflammatory stimuli (García-Posadas et al., 2022), alongside a well-established ocular guinea pig animal model *in vivo* (Rank and Whittum-Hudson, 1994; Filipovic et al., 2017).

Consistent with our earlier findings in genital A2EN epithelial cells (Pfundner et al., 2025), DHP/Alg significantly reduced the number of chlamydial inclusions in ocular IM-HConEpiC cells *in vitro* in a dose-dependent manner. These effects were observed at concentrations that did not affect cell viability, indicating that the formulation is well tolerated. Importantly, neither DHP nor alginate alone had a measurable effect, indicating that anti-chlamydial activity depends on the combined formulation rather than on its individual components. Given that chlamydial infection critically depends on elementary body adhesion to epithelial surfaces and subsequent entry, processes mediated in part by interactions with sulfated glycosaminoglycans such as heparan sulfate (Zhang and Stephens, 1992; Elwell et al., 2016), this effect may reflect modulation of the host-pathogen interface. While DHP may interact with bacteria (Li et al., 2023) and alginate may alter the epithelial surface (Augst et al., 2006), their combination may influence early host-pathogen interactions, including attachment and initial stages of entry, which are known to be sensitive to disruption of surface interactions (Elwell et al., 2016).

In contrast, this *in vitro* activity, consistently observed across both genital (Pfundner et al., 2025) and ocular epithelial cell models, did not translate into a measurable reduction in chlamydial burden *in vivo*. Neither qPCR nor culture-based quantification of chlamydial IFUs revealed significant differences between treated and untreated animals. This discrepancy highlights the challenges associated with translating *in vitro* antimicrobial activity into measurable *in vivo* efficacy. It underscores the importance of testing antimicrobial strategies in physiologically relevant systems, where multiple factors shape treatment outcomes. This is particularly relevant for biomaterial-based topical approaches, where local retention and effective tissue exposure are critical determinants of biological activity.

Factors such as limited retention, tissue barriers, and the local microenvironment can substantially reduce effective exposure (Scannell et al., 2012). The ocular surface, in particular, presents additional constraints, including tear turnover, mucus, and immune components, which may further limit local concentration (Gaudana et al., 2010; Willcox, 2019). In addition, the timing of treatment relative to infection may be critical, as DHP/Alg appears to act primarily during early stages of infection (Pfundner et al., 2025). Therefore, it is important to note that treatment was initiated at an early stage of infection (2h post-infection), which does not fully reflect clinical practice, where treatment is typically administered after the onset of symptoms. However, topical administration was continued daily for 14 days to cover both the early and established phases of infection. While early intervention may contribute to the observed effects, this design also allows assessment of treatment during ongoing infection. Nevertheless, future studies should evaluate delayed treatment initiation to better mimic clinical scenarios. 

Importantly, although the guinea pig is one of the most relevant and well-established models for ocular chlamydial infection and trachoma-like pathology, the availability of species-specific immunological reagents and consumables for this model remains limited compared to those for mouse and human systems. In particular, access to validated anti-guinea pig antibodies for detailed immune profiling remains highly restricted, a well-recognized challenge in the field. Consequently, while the present study supports the possibility that DHP/Alg may contribute to differences in ocular pathology independently of measurable bacterial reduction, more detailed mechanistic analyses will require broader availability of guinea pig-specific immunological tools in future studies.

Methodologically, qPCR and IFU-based quantification yielded different absolute values, as expected, with qPCR detecting total chlamydial DNA and IFU measurements reflecting only viable organisms. Despite these differences, both methods showed consistent relative results across groups, supporting the conclusion that DHP/Alg treatment did not reduce bacterial burden *in vivo*. Several factors may contribute to the lack of measurable *in vivo* efficacy.

The concentration used *in vivo* (75 µg/mL) was selected as a pragmatic starting point for topical administration under repeated dosing conditions, based on prior tolerability data from both ocular and genital epithelial models, to ensure biological compatibility across systems. While higher concentrations (up to 200 µg/mL) demonstrated increased efficacy and were well tolerated in the ocular *in vitro* model, they were cytotoxic in the genital epithelial system in our previous work (Weng et al., 2017). In addition, lower concentrations were preferred for repeated ocular administration (14 consecutive days) to minimize exposure to DMSO. Therefore, the *in vivo* dose was chosen as a compromise rather than to maximize *in vitro* efficacy (Pfundner et al., 2025).

Importantly, because we did not evaluate dose escalation *in vivo*, we cannot exclude the possibility that higher concentrations may have led to improved outcomes. However, given the rapid tear turnover and clearance at the ocular surface, increasing concentration alone is unlikely to overcome delivery constraints without simultaneous optimization of formulation properties and retention. In this context, suboptimal local exposure, further influenced by factors such as compound stability, distribution, and retention, may have contributed to the absence of a measurable antimicrobial effect *in vivo* (Gaudana et al., 2010).

Interestingly, despite the absence of a measurable reduction in bacterial load, DHP/Alg treatment was associated with a transient improvement in pathology in the high-dose infection group, particularly on day 7 post-infection. This observation raises the possibility that the formulation may influence disease outcome independently of direct bacterial clearance; however, this hypothesis requires further investigation and direct experimental validation. Lignin and lignin-derived biomaterials are known for their antioxidant, anti-inflammatory, and tissue-supportive properties (Zmejkoski et al., 2018). Rather than functioning as classical antimicrobials, such formulations have been shown to influence oxidative stress and promote epithelial repair. Although the present study was not designed to investigate these mechanisms directly, the observed findings are compatible with this framework.

Histopathological assessment was performed in a blinded manner using representative conjunctival tissue sections; however, formal quantitative histopathological scoring was not performed in this exploratory study. This limitation should be considered when interpreting the histological findings. Nevertheless, histological evaluation generally showed reduced inflammatory cell infiltration and better preservation of conjunctival architecture in DHP/Alg-treated animals on day 7 post-infection compared with untreated controls. These observations may indicate reduced infection-induced tissue damage rather than strong direct suppression of bacterial replication. Under conditions of pronounced inflammatory stress, such differences may become more readily detectable, whereas at lower infection burdens, baseline pathology may be insufficient to reveal measurable histological differences between groups.

A limitation of the present study is the absence of an *in vivo* antibiotic control group, which would have enabled a direct assessment of treatment efficacy against a clinically established intervention while also reducing animal use in accordance with the 3Rs principle. While the current study focused on evaluating the intrinsic activity of the DHP/Alg formulation relative to untreated controls, including such a comparator will be important in future studies, particularly for formulation optimization and translational evaluation.

Another important limitation is the likely short residence time of the formulation on the ocular surface, which may reduce overall treatment efficacy. The ocular environment is characterized by rapid tear turnover and efficient clearance mechanisms, posing a major challenge for topical drug delivery. In this context, the discrepancy between *in vitro* efficacy and *in vivo* performance may, at least in part, be explained by insufficient local exposure. Ongoing work in our group, therefore, focuses on optimizing formulation strategies to enhance ocular retention and delivery. In particular, we are exploring thermosensitive systems, such as poloxamer-based (Longo et al., 2025), as well as mucoadhesive approaches, including chitosan-based formulations (Schuerer et al., 2018), to improve stability, prolong residence time, and increase local bioavailability.

## 5 Conclusion

Taken together, our results are consistent with a model in which DHP/Alg acts at the pathogen-host interface, with context-dependent effects that are more pronounced under conditions of high inflammatory burden.

While alternative antimicrobial strategies are gaining increasing attention, their role should be considered alongside existing therapeutic options. In the case of *C. trachomatis*, where highly effective antibiotics are available (Evans et al., 2019), and no clinically significant antimicrobial resistance has been observed to date (O'Brien et al., 2019), the goal is not to replace established treatments but rather to complement them. However, the absence of resistance in *C. trachomatis* should not lead to complacency, particularly given the widespread and repeated use of antibiotics in endemic settings. Notably, tetracycline-resistant strains have been described in *Chlamydia suis*, demonstrating that Chlamydiae can acquire resistance under selective pressure (Unterweger et al., 2020), and *in vitro* studies further support the potential for the development of resistance under antibiotic exposure (Choi et al., 2024).

Locally acting, non-antibiotic approaches may help reduce treatment burden, limit systemic exposure, and potentially mitigate future selection pressure associated with repeated antibiotic use. Our results suggest that, rather than acting as a classical antimicrobial, DHP/Alg may primarily reduce infection-induced tissue damage *in vivo*, with protective effects most apparent under conditions of pronounced inflammatory stress. Future studies should focus on optimizing formulation, dosing, and timing of administration, particularly in relation to early infection events, while further clarifying the mechanisms underlying its effects on ocular pathology. These efforts will be critical to fully defining the potential of DHP/Alg as a complementary strategy for managing chlamydial infections.

## Data Availability

The raw data supporting the conclusions of this article will be made available by the authors, without undue reservation.

## Supplementary material

Supplementary Figure 1 Analysis of ocular swab samples collected from guinea pigs infected with 10^6^, 10^4^, or 10^2^ IFU/eye of *C. caviae* and either left untreated or treated with DHP/Alg (75 µg/mL) during the post-infection period. Infectious burden in each graph is expressed relative to the mean burden of untreated animals at day 4 post-infection with the respective dose of *C. caviae* (10^6^, 10^4^, or 10² IFUs). **(A)** Quantification of infectious units in ocular swab suspensions collected at days 4, 7, and 14 post-infection, as determined by fluorescence microscopy. IFU/mL values were calculated by counting inclusions in 20 microscopic fields, extrapolating to the total coverslip area, and correcting for the dilution factor. **(B)** Quantification of chlamydial burden in corresponding swab samples by qPCR. Genome copy numbers were determined using a standard curve and converted from copies per reaction well to genome copies/mL of swab suspension. Statistical significance was assessed using two-way ANOVA followed by Sidak’s multiple comparisons test. Significance levels are indicated as *p ≤ 0.05, **p ≤ 0.01, and ***p ≤ 0.001.
