# Supplementary figures and images for: Lignin/alginate biomaterials as a promising complementary approach in ocular chlamydial infection
Source: Front Cell Infect Microbiol. 2026 Jun 29;16:1843021. doi: 10.3389/fcimb.2026.1843021 (PMC13357531; doi:10.3389/fcimb.2026.1843021)

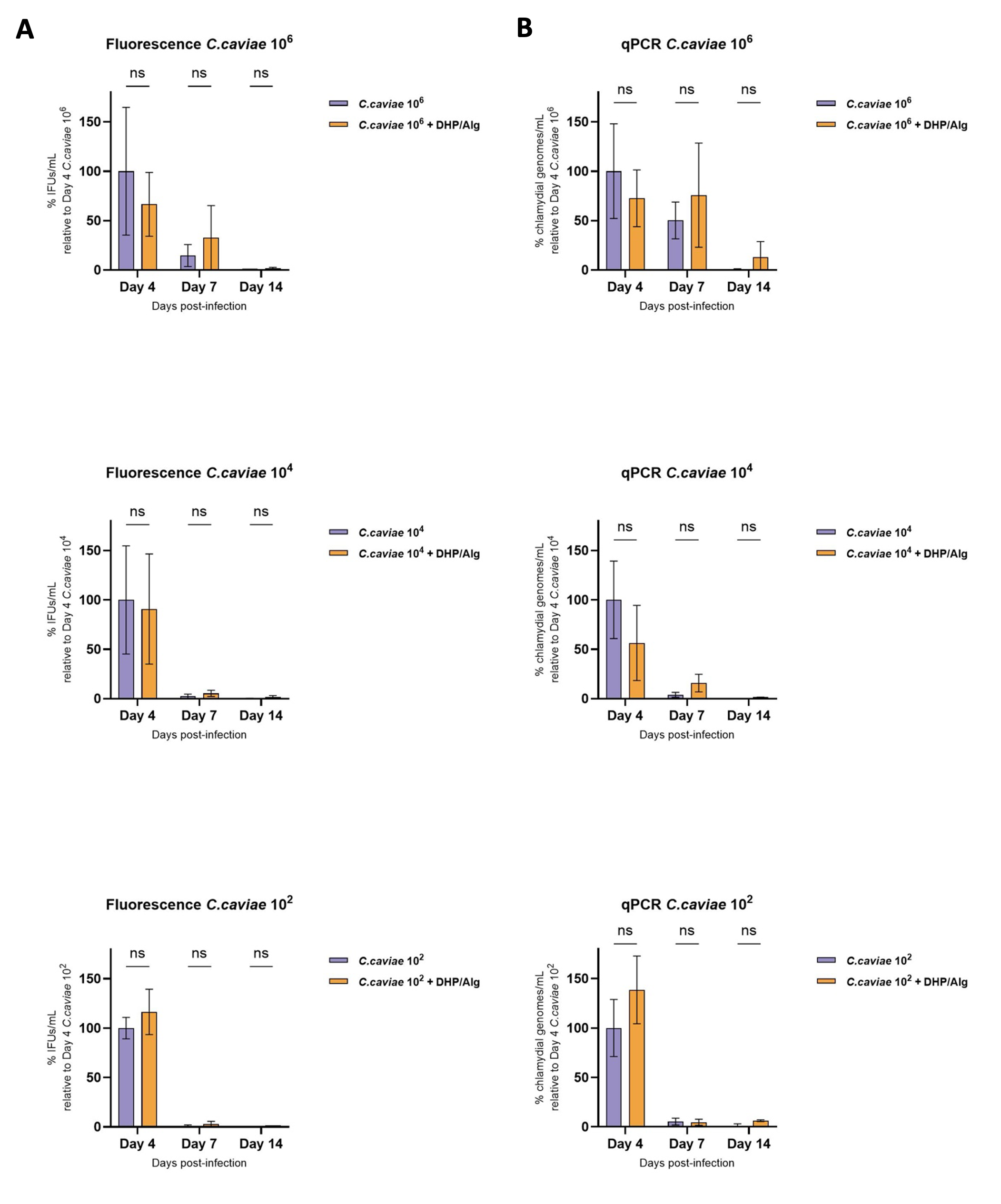

Supplement: Supplementary Figure 1 — Analysis of ocular swab samples collected from guinea pigs infected with 106, 104, or 102 IFU/eye of C. caviae and either left untreated or treated with DHP/Alg (75 µg/mL) during the post-infection period. Infectious burden in each graph is expressed relative to the mean burden of untreated animals at day 4 post-infection with the respective dose of C. caviae (106, 104, or 10² IFUs). (A) Quantification of infectious units in ocular swab suspensions collected at days 4, 7, and 14 post-infection, as determined by fluorescence microscopy. IFU/mL values were calculated by counting inclusions in 20 microscopic fields, extrapolating to the total coverslip area, and correcting for the dilution factor. (B) Quantification of chlamydial burden in corresponding swab samples by qPCR. Genome copy numbers were determined using a standard curve and converted from copies per reaction well to genome copies/mL of swab suspension. Statistical significance was assessed using two-way ANOVA followed by Sidak’s multiple comparisons test. Significance levels are indicated as *p ≤ 0.05, **p ≤ 0.01, and ***p ≤ 0.001. [file Image1.jpeg]
